# Strategies to Screen for Diabetic Retinopathy in Chinese Patients with Newly Diagnosed Type 2 Diabetes: A Cost-Effectiveness Analysis

**DOI:** 10.1097/MD.0000000000001989

**Published:** 2015-11-13

**Authors:** Bin Wu, Jin Li, Haixiang Wu

**Affiliations:** From the Medical Decision and Economic Group, Department of Pharmacy (BW); Department of Ophthalmology, Ren Ji Hospital, affiliated with the School of Medicine, Shanghai Jiaotong University, Shanghai (JL); and Department of Ophthalmology, Eye & ENT Hospital of Fudan University, Shanghai, China (HW).

## Abstract

To investigate the cost-effectiveness of different screening intervals for diabetic retinopathy (DR) in Chinese patients with newly diagnosed type 2 diabetes mellitus (T2DM).

Chinese healthcare system.

Chinese general clinical setting.

A cost-effectiveness model was developed to simulate the disease course of Chinese population with newly diagnosed with diabetes. Different DR screening programs were modeled to project economic outcomes. To develop the economic model, we calibrated the progression rates of DR that fit Chinese epidemiologic data derived from the published literature. Costs were estimated from the perspective of the Chinese healthcare system, and the analysis was run over a lifetime horizon. One-way and probabilistic sensitivity analyses were performed. Total costs, vision outcomes, costs per quality-adjusted life year (QALY), the incremental cost-effectiveness ratio (ICER) of screening strategies compared to no screening.

DR screening is effective in Chinese patients with newly diagnosed T2DM, and screen strategies with ≥4-year intervals were cost-effective (ICER <$7,485 per QALY) compared to no screening. Screening every 4 years produced the greatest increase in QALYs (11.066) among the cost-effective strategies. The screening intervals could be varied dramatically by age at T2DM diagnosis. Probabilistic sensitivity analyses demonstrated the consistency and robustness of the cost-effectiveness of the 4-year interval screening strategy.

The findings suggest that a 4-year interval screening strategy is likely to be more cost-effective than screening every 1 to 3 years in comparison with no screening in the Chinese setting. The screening intervals might be tailored according to the age at T2DM diagnosis.

## Abstract 2

Supplemental Digital Content is available in the text

## INTRODUCTION

Diabetic retinopathy (DR) is one of the most frequent causes of blindness in the population aged 20 to 74 years and is characterized by angiogenesis in the retina.^1^ As the global prevalence of diabetes mellitus (DM) continues to increase, DR remains the leading cause of blindness in many developed countries. One-third of DM patients exhibit signs of DR, and one-third of these patients exhibit vision-threatening retinopathy.^2,3^ The reported prevalence of DR, nonproliferative diabetic retinopathy (NPDR), and proliferative diabetic retinopathy (PDR) in the Chinese general and diabetic population were 1.3% and 23%, 1.1% and 19.1%, and 0.1% and 2.8%,^4^ respectively. Macular edema (ME) was present in 5.2%, and clinically significant ME was present in 3.5% of patients with DM.^5^ However, many Chinese diabetic patients with DR have not been diagnosed and do not receive appropriate interventions.^6^

Because laser photocoagulation can reduce the risks of vision loss in patients with PDR and ME, DR screening is recommended upon the diagnosis of diabetes and either yearly or every second year thereafter in people with type 2 diabetes.^2^ Economic evaluations suggest that systematic screening for DR is cost-effective with respect to sight years preserved compared with no screening.^7^ According to the diagnosis and treatment guidelines for DR in China, DR should be screened annually.^8^ To the best of our knowledge, a comprehensive economic evaluation of a screening strategy for DR in the Chinese mainland setting has not been conducted. Because most published economic analyses concerning DR screening come from developed countries, the economic outcomes of systematic screening for DR need to be carefully investigated in the unique health environment of China. The object of the present study is to evaluate the relative clinical benefits and cost-effectiveness of potential screening intervals in a cohort of Chinese patients with newly diagnosed type 2 diabetes mellitus (T2DM).

## METHODS

### Analytic Overview

Public health policy usually implemented over a long period of time. However, the follow-up time of clinical trials could rarely track lifetime course of the disease, and observational data may has limited value in predicting the future impact of a proposed policy. Thus, mathematical modeling techniques would be used to supply decision making information in the current analysis, which plays an increasingly important role in helping to guide the policy decision making.^9^ A discrete event simulation (DES) policy model was developed to measure the economic and health outcomes of DR screening. The main reason that a DES was selected is that this model can closely replicates the disease course with the more powerful flexibility in handling perspectives and structural variations with few restrictions in comparison in comparison with the decision trees and Markov models.^10^ When the model begin to simulate, we created hypothetical patients with specific characteristics, which was then duplicated to generate several identical cohorts for assessing the different strategies. All of the potential risks and events would be incurred by patients during the course of disease simulation. The event with the shortest time of arrival would be chosen as the occurred event. The time of arrival for each type of event was randomly sampled based on the statistical distribution of happening time. Once the event arrived, the attributes of patients would then be renewed instantaneously for recalculating the risks and event times.

**FIGURE 1:**
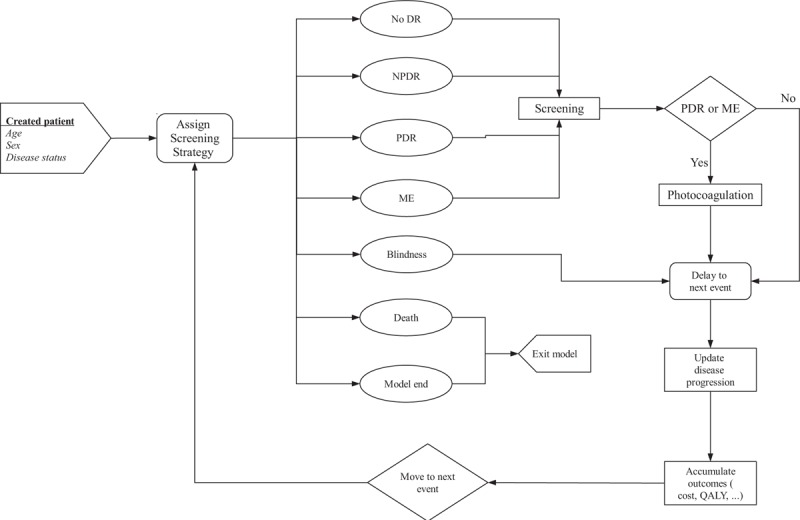
General simulation process of DR in patients with type 2 diabetes. Patients’ health state could change if an event (eg, NPDR) occurred. The risks depended on the underlying disease and the treatment strategy received. DR = diabetic retinopathy; NPDR = nonproliferative diabetic retinopathy; PDR = proliferative diabetic retinopathy; ME = macular edema.

The patients who were included in the model at baseline reflected the characteristics of Chinese patients with type 2 diabetes,^1,5,11,12^ including the age, sex, and disease status of DR at the diagnosis time point. A hypothetical patient population with newly diagnosed type 2 diabetes was created for this simulation. Each simulated patient in the cohort was assigned specific characteristics and then cloned to receive one of the following screening strategies: no DR screening or screening on 1-, 2-, 3-, 4-, 5-year interval basis. The screening strategies was chosen based on the previous studies, which showed the screening interval varied from 1 to 5 years.^7^ We assumed that patients with diagnosed NPDR, PDR, or ME were subsequently referred for annual examination. Patients with confirmed PDR or ME were treated with laser photocoagulation treatment. Risks for disease progression related to treatment were then assigned to each patient. Each patient could be subjected to the following health events as shown in Figure 1: no DR, NPDR, PDR, ME, and blind from DR (bilateral best-corrected VA <6/60).

The primary model outputs would capture life years (LYs), quality-adjusted life years (QALYs), and the direct medical consumption, and a cost-utility analysis was performed,^13^ where incremental cost-effectiveness ratios (ICERs) were calculated by the difference in costs between strategies divided difference in their effect.^14^ The model outcomes were measured based on 100 iterations comprising a cohort of 100,000 patients until their death or 100 years old (lifetime), which was used as the time horizon of the model because diabetes and DR were lifetime diseases. Future costs and QALY outcomes were annually discounted at a rate of 3% according to the health economic evaluation guideline in China.^15,16^ This economic study was based on a literature review and model techniques, and did not require approval by the institutional Research Ethics Board.

### Clinical Data

Due to the absence of relevant epidemiological studies, we cannot directly estimate the risks of NPDR, PDR, and ME in Chinese patients with type 2 diabetes. We used the calibration approach, which is a process of producing model output parameters that best predict observed data, to identify a series of good fitting parameter sets by the genetic algorithm that provided model predictions that were consistent with the observed Chinese prevalence of NPDR, PDR, and ME.^17,18^ Additional details regarding the methods used for model calibration and validation are explained in the Appendix, http://links.lww.com/MD/A519. Calibration targets were derived by conducting a literature review in PubMed, EMBASE, and the China National Knowledge Infrastructure database. The prevalences of NPDR, PDR, and ME were collected and are presented in Appendix, http://links.lww.com/MD/A519.

### Mortality

Natural mortality could occur at any point during the disease course. The model used a normal life table from the life tables for the World Health Organization's (WHO) member states (2011) to adjust the mortality multiplier for patients with diabetes and DR (Table 1).^21,31^

**TABLE 1:**
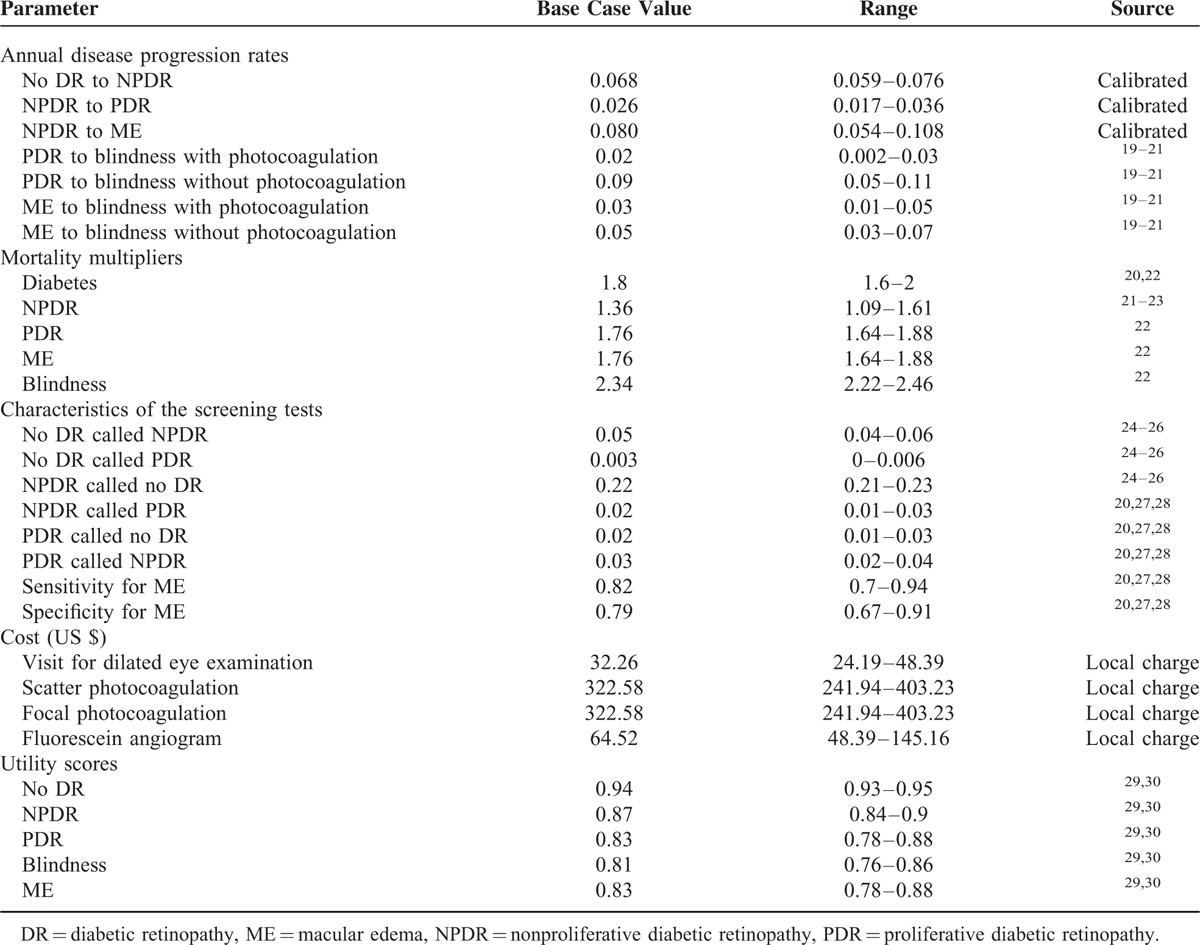
Parameter Values for the Model

### Cost and Utility Data

Costs were estimated from the perspective of the Chinese healthcare system in the general clinical setting and are reported in 2014 US dollar equivalents  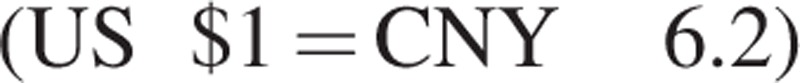. The following direct medical cost components were considered: the costs of DR screening, drugs, regular clinic fees, laser photocoagulation (focal and scatter), and fluorescein angiogram. All unit costs of the health resources were estimated using data from the local health system or the National Development and Reform Commission (NDRC) of China.^32^

The utility scores related to DR, including no DR, NPDR, PDR, ME, and blindness, were derived from relevant published studies.^29,30^ A total of 406 eligible T2DM patients with DR in Chinese Taiwan were recruited for measuring utility values by using time trade-off method.^29^

### Sensitivity Analysis

To test the robustness of the model, 1- and 2-way sensitivity analyses of the parameters were conducted in the decision model over the estimate ranges presented in Table 1. We performed probability sensitivity analyses (PSAs) in which uncertainties across all of the variables were varied simultaneously for 1000 iterations across 95% confidence intervals. In cases in which these confidence intervals were not available, we used plausible (eg, ±25%) values.^33^ Triangular distributions (for costs) and beta distributions (for probabilities and utilities) were used. Cost-effectiveness acceptability curves were constructed to summarize the uncertainty of the cost-effectiveness estimates in the context of a broader range for willingness-to-pay per QALY. In accordance with the WHO recommendation,^34–36^ the per capita GDP value of China in 2014 ($7485) was used as the cost-effectiveness threshold.

## RESULTS

### Cost-Effectiveness Analysis

Our model estimated the costs and health outcomes of the different strategies (Table 2). Strategies with longer screening intervals resulted in lower costs and less effectiveness. In comparison with no screening, the ICERs were lower than the threshold of $7485 when the screening intervals were greater than 3 years. Increasing to annual or biennial screening yielded increased QALYs and less time affected by blindness, but the marginal cost caused the ICERs to exceed the threshold.

**TABLE 2:**
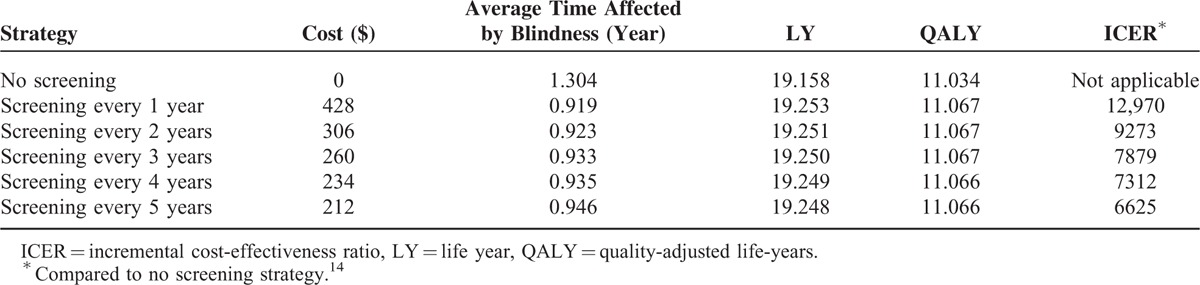
Cost-Effectiveness of Different Diabetic Retinopathy Screening Intervals

### Sensitivity Analysis

**FIGURE 2:**
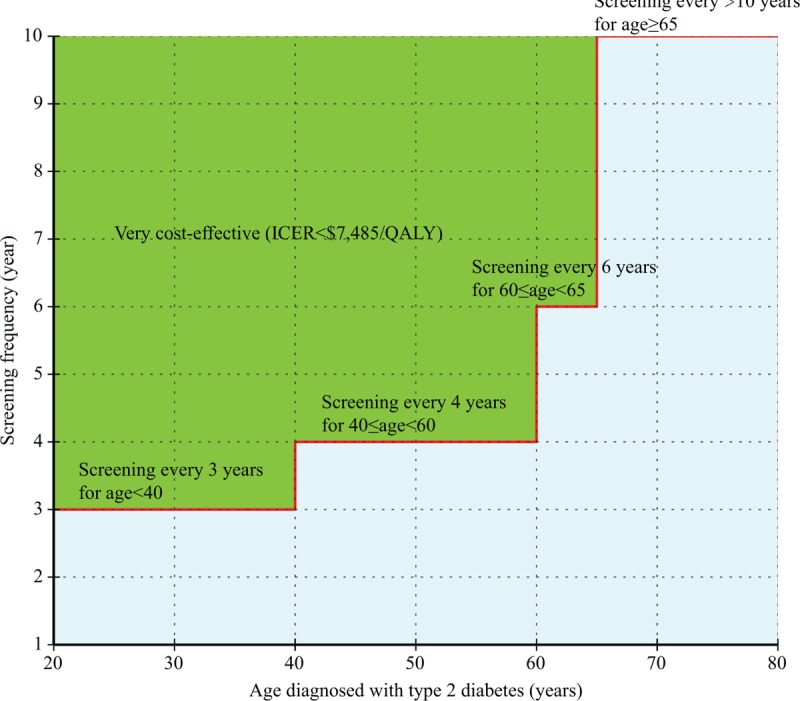
Impact of the age diagnosed with type 2 diabetes on the screening frequency. The step red solid line indicates the very cost-effective screening strategy.

Figure 2 shows that screening patients who were diagnosed with T2DM at age <40, ≥40 age <60 and ≥60 age <65 years every 3, 4, and 6 years, respectively, would be cost-effective. Individuals diagnosed at age >65 could be screened every >10 years.

**FIGURE 3:**
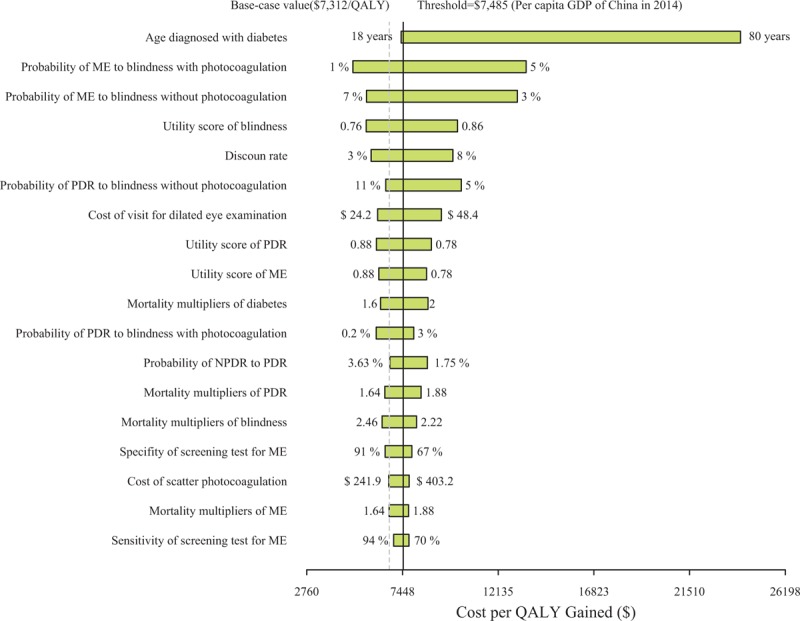
Tornado diagram representing the cost per QALY gained in 1-way sensitivity analysis for screening every 4 years versus no screening. The width of the bars represents the range of the results when the variables were changed. The vertical dotted and solid line represents the base case results and threshold, respectively. DR = diabetic retinopathy; NPDR = nonproliferative diabetic retinopathy; PDR = proliferative diabetic retinopathy; ME = macular edema.

The 1-way sensitivity analyses revealed that some model variables had a substantial impact on the results; these variables are presented in the tornado graphs in Figure 3. The most influential variables were the age diagnosed with type 2 diabetes, followed by the probability of ME to blindness with and without photocoagulation. Other parameters, including the 3 calibrated parameters, had little to moderate effects on the model outputs.

**FIGURE 4:**
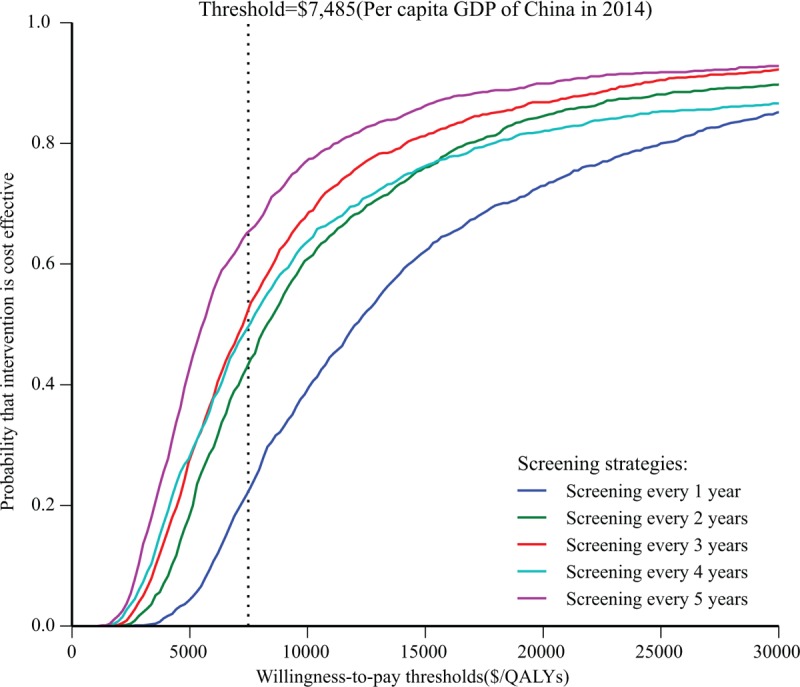
Cost-effectiveness acceptability curves for the different screening strategies compared to no screening.

Figure 4 shows that screening every 4 or 5 years could achieve over one-half the likelihood of cost-effectiveness compared to no screening at a threshold level of per capita GDP of China in 2014 ($7485).

## DISCUSSION

To the best of our knowledge, this is the first economic evaluation of DR screening in China. Our study indicates that annual screening offers a paucity of health benefits compared to other screening intervals. According to the WHO recommendation for the cost-effectiveness threshold, >3-year screening strategies are very cost-effective for a typical newly diagnosed patient in China because the ICERs of such patients are lower than the threshold of $7485 per additional QALY gained (which represents three times the per capita GDP of China in 2014).^34–36^ In particular, the ICER of the 4-year screening strategy produced the greatest health outcomes relative to other screening strategies at more than 4-year screening intervals. These findings suggest that 4-year screening intervals may be a cost-effective alternative approach in the Chinese setting when costs and outcomes are considered.

The aggregated evidence from both the natural history and cost-effectiveness models favors a screening interval >1 year but ≤2 years.^37^ However, these studies were conducted in high-income areas, and it is difficult to generalize the results to low- and middle-income areas.^38^ The cost-effectiveness analysis from high-income countries revealed that annual retinal screening for all patients with T2DM without previously detected retinopathy may not be warranted on the basis of cost-effectiveness^20^; these findings are similar to those of our research, although the economic setting differs. One economic evaluation from the Chinese Taiwan area found that annual screening for DR among Chinese patients with type 2 diabetes should be cost-effective over a 10-year time horizon because efficacy and utility decreased while cost increased with the length of the screening/surveillance interval.^6^ However, the analysis did not note the increasing surveillance intervals once DR is detected through screening.^37^ Moreover, unlike most models, the authors did not consider mortality and the impact of DR on all-cause mortality, which may factor into the costs. Recently, 1 economic study from India reported that a 1-off DR telescreening program is cost-effective compared with no screening in the setting of rural India.^39^ The reason for the difference might be that the cost-effective threshold in India is $1320/QALY, which is nearly one-sixth the threshold in China ($7485/QALY).

We performed extensive sensitivity analyses to examine the robustness of model outcomes. The age of patients with newly diagnosed T2DM was the most influential factor for clinical and economic outcomes. When the age of newly diagnosed T2DM patients increased, the cost-effectiveness of screening every four years decreased. As shown in Figure 2, for younger patients (diagnosed at age < 40 years), screening every 3 years was cost-effective. However, for older patients, screening is only necessary every 6 (≥60 diagnosed age <65 years) or >10 years (diagnosed age ≥65 years). These findings suggest that tailoring the screening interval according patient age could improve the cost-effectiveness of DR screening. These reports also determined that DR screening of younger patients exhibited long-term cost-effectiveness compared to no screening.^20,21^ On the basis of the current clinical trial and from the perspective of the Chinese healthcare system, the results of these studies are consistent with ours.

The results of this analysis must be interpreted carefully given the limitations of the data and study design. First, some of the probability estimates that were employed were obtained by the calibration method and, thus, do not avoid uncertainty although they were comparable with other published study.^21^ Sensitivity analyses demonstrate that some of these parameters exhibit a moderate impact on the cost-effectiveness of a screening strategy. Second, in the current analyses, because of the absence of relevant epidemiological studies in China, the risks of developing DR were not stratified by the risk factors, such as glycemic control, lipid levels, and blood pressure,^40^ and some of the parameter values were derived from literature that was published abroad and thus may not reflect Chinese data. Third, recent studies have revealed that vascular endothelial growth factor inhibitors can affect vision with center-involved diabetic macular edema (DME)^41^; nevertheless, we did not take this issue into account in our current model because these inhibitors are not widely prescribed in China. Fourth, we did not measure the additional benefits of an annual screening strategy, such as the early detection and intervention of glaucoma and cataracts. Finally, the results obtained from this analysis apply only to a narrow patient cohort: patients with newly diagnosed T2DM without DR. Nonetheless, because the results of this analysis reflect the clinical conditions of DR screening that are common in China, we believe that the results can serve as important reference points for Chinese decision-makers.

In conclusion, our study suggests that a 4-year screening program for DR is cost-effective compared to no screening in the Chinese setting because little benefit was achieved by 1- to 3-year screening. Varying screening frequencies might be tailored according to patient age; we believe that this focus will provide interesting insights into how to best reduce the disease burden associated with DR in China.
